# Supplementary material for: AGC/AKT Protein Kinase SCH9 Is Critical to Pathogenic Development and Overwintering Survival in Magnaporthe oryzae
Source: J Fungi (Basel). 2022 Jul 31;8(8):810. doi: 10.3390/jof8080810 (PMC9410157; doi:10.3390/jof8080810)
Supplement: Supplementary file 1 [file jof-08-00810-s001.zip › Supplementary figures and table legends.pdf]

AGC/AKT protein kinase SCH9 is critical to pathogenic development and overwintering survival in *Magnaporthe oryzae*

Wajjiha Batool[1], Liu Chang[1], Fan Xiaoning[1], Penghui Zhang[1], Hu Yan[1], Wei Yi[1], Zhang Shi-Hong[1]*

[1] The Key Laboratory for Extreme-environmental Microbiology, Liaoning province, College of Plant Protection, Shenyang Agricultural University, Shenyang, 110866, China.

Figure S1. Protein 3D structure, Localization, Pathway, Gel and qPCR results for MoSch9 mutants' confirmation **(A)** MoSCH9 protein 3D structure showing conserved ATP binding and active site. **(B)** Nuclear localization prediction of MoSch9 using ProtComp. **(C)** Schematic presentation of MoSch9 targeted disruption using homologs recombination approach. **(D)** PCR Gel results confirming the targeted replacement of MoSch9 with hygromycin gene. **(E)** Expression pattern of MoSch9 in MoSch9 deleted strains (Δ*Mosch9-33*, Δ*Mosch9-41*) taking MoSch9 expression in wild type (Y34) as a control (=1). Error bars indicate the means ± SD calculated from three independent technical replicates in which triplicate biological samples for each strain were examined in each experiment.

Figure S2: Impact of MoSch9 gene deletion on spore mediated pathogenicity, appressorium formation, and penetration ability of *M. oryzae.* Displays the conidia-mediated virulence capabilities of the Δ*Mosch9* strains compared to wild-type strains on **(A)** detached intact and injured barley leaves and **(B)** 3 weeks old susceptible rice seedlings. **(C)** Comparative conidial germination and appressorium formation by Δ*Mosch9*, and wild-type strains on hydrophobic slides. **(D)** Shows statistical record obtained from microscopic study of the % germ tube and appressorium formed by wild-type and Δ*Mosch9* spores on hydrophobic slides at 4 and 8 hpi. One-way ANOVAs (non-parametric) was performed in GraphPad Prism 7 and Microsoft Excel **(E)** Histopathological photograph representing comparative spore mediated penetration and invasive growth efficacy of Δ*MoSch9* mutants' strain and wild type on barley leaves. Scale=20 µm **(F)**. Displays the hyphal and conidia-mediated virulence capabilities of the complementation strain on detached intact and injured barley leaves and rice leaves. **(G)** Growth of complementation strain on CM, CM with different stress induced chemicals and on RBM at different temperature. MoSch9 mutants' virulence and stress growth defects were restored in the complementation strain.

Figure S3: Differentially expressed genes after ΔMosch9 deletion **(A)**. Displays heat map showing differentially expressed genes of wild type and MoSch9 mutant at different temperatures (33°C, 10°C, and 25°C). **(B)** Graphical representation of down regulated and up regulated genes of ΔMosch9 vs wild type at different temperatures. **(C)** Venn diagram representing common genes that were differentially expressed during all three temperatures. **(D)** KEGG enrichment analysis of differentially expressed genes at 10°C.

Table S1: (a). SCH9 functionally characterized in different fungal class. **(b)** SCH9 used for phylogenetic analysis (**c**) Primers used in this study.
