# Supplementary figures and images for: AGC/AKT Protein Kinase SCH9 Is Critical to Pathogenic Development and Overwintering Survival in Magnaporthe oryzae
Source: J Fungi (Basel). 2022 Jul 31;8(8):810. doi: 10.3390/jof8080810 (PMC9410157; doi:10.3390/jof8080810)

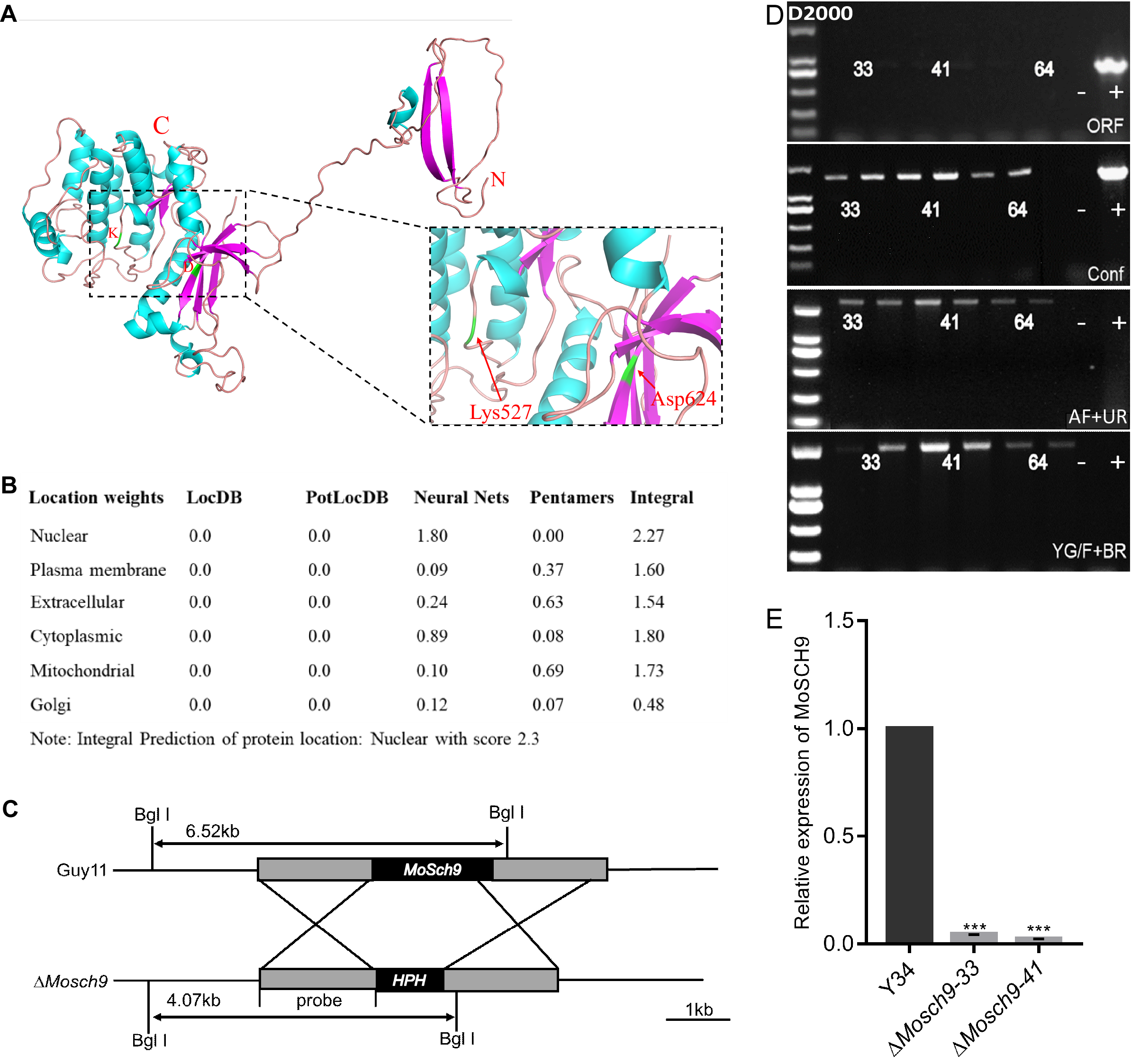

Supplement: Supplementary file 1 [file jof-08-00810-s001.zip › fig s1.tif]

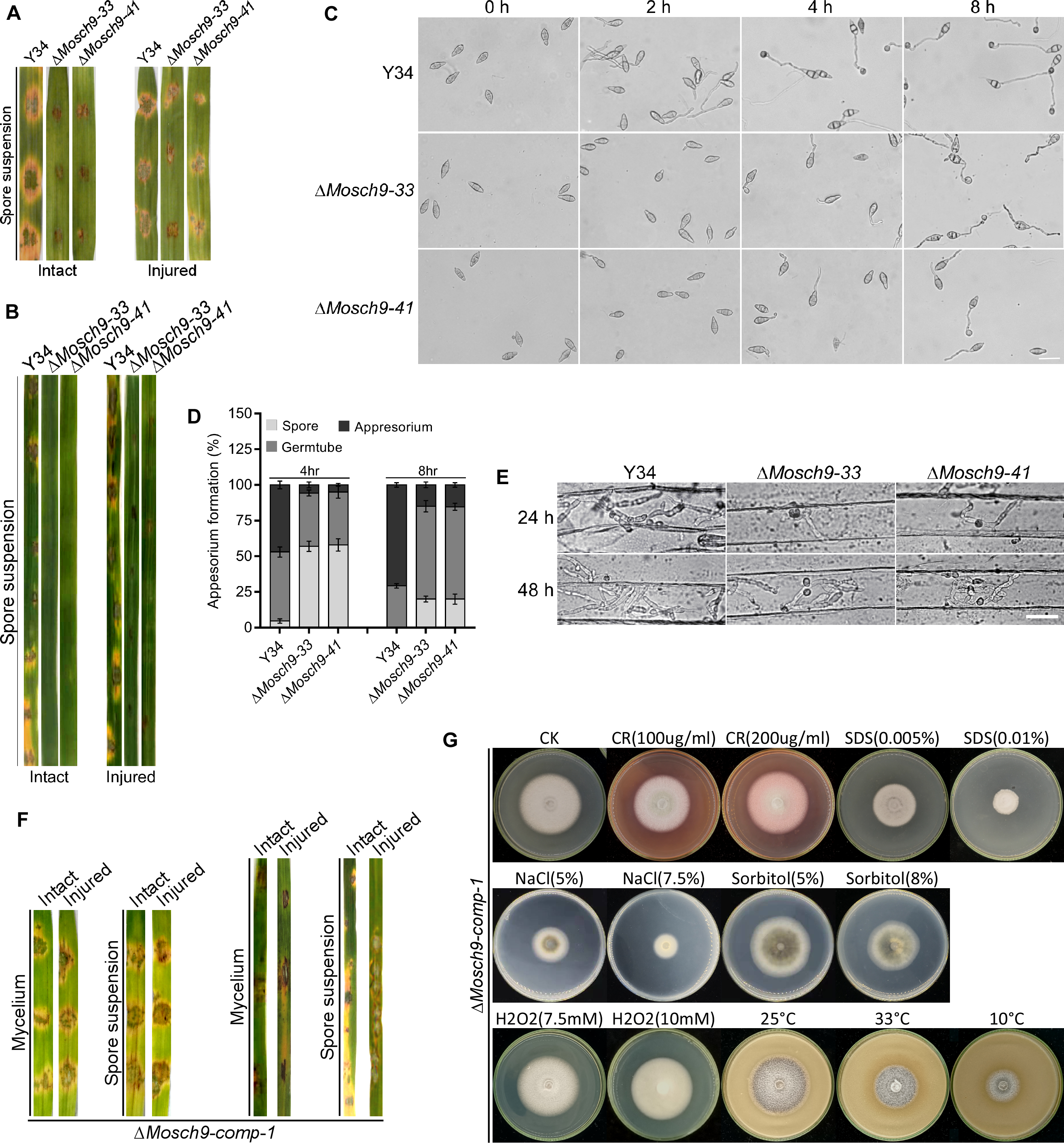

Supplement: Supplementary file 1 [file jof-08-00810-s001.zip › fig s2.tif]

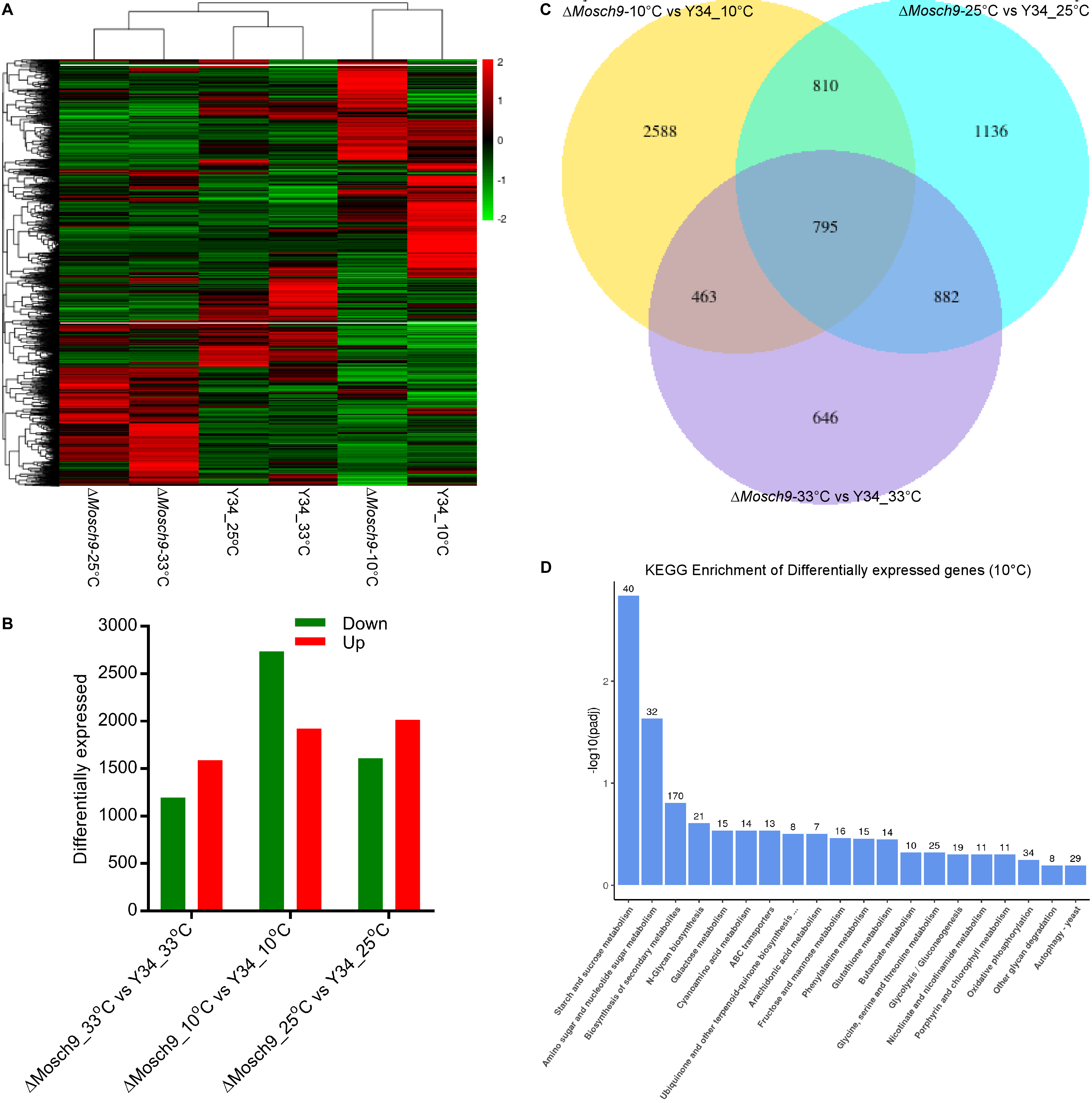

Supplement: Supplementary file 1 [file jof-08-00810-s001.zip › fig s3.tif]
